# Fast Production of Cellulose Nanocrystals by Hydrolytic-Oxidative Microwave-Assisted Treatment

**DOI:** 10.3390/polym12010068

**Published:** 2020-01-02

**Authors:** Luana Amoroso, Giuseppe Muratore, Marco Aldo Ortenzi, Stefano Gazzotti, Sara Limbo, Luciano Piergiovanni

**Affiliations:** 1 Department of Agricultural, Food and Environment (Di3A), Università degli Studi di Catania, Via Santa Sofia 100, 95123 Catania, Italy; luana.amoroso@unict.it (L.A.); g.muratore@unict.it (G.M.); 2 CRC Laboratorio di Materiali e Polimeri (LaMPo), Department of Chemistry, Università degli Studi di Milano, Via Golgi 19, 20133 Milano, Italy; Marco.Ortenzi@unimi.it (M.A.O.); Stefano.Gazzotti@unimi.it (S.G.); 3 DeFENS, Department of Food, Environmental and Nutritional Sciences—PackLAB Università degli Studi di Milano, Via Celoria 2, 20133 Milano, Italy; sara.limbo@unimi.it

**Keywords:** microwave technology, cellulose nanocrystals, energy-saving, fast-production

## Abstract

In contrast to conventional approaches, which are considered to be energy- and time-intensive, expensive, and not green, herein, we report an alternative microwave-assisted ammonium persulfate (APS) method for cellulose nanocrystals (CNCs) production, under pressurized conditions in a closed reaction system. The aim was to optimize the hydrolytic-oxidative patented procedure (US 8,900,706), replacing the conventional heating with a faster process that would allow the industrial scale production of the nanomaterial and make it more appealing to a green economy. A microwave-assisted process was performed according to different time–temperature programs, varying the ramp (from 5 to 40 min) and the hold heating time (from 60 to 90 min), at a fixed reagent concentration and weight ratio of the raw material/APS solution. Differences in composition, structure, and morphology of the nanocrystals, arising from traditional and microwave methods, were studied by several techniques (TEM, Fourier transform infrared spectroscopy (FTIR)-attenuated total reflectance (ATR), dynamic light scattering (DLS), electrophoretic light scattering (ELS), thermogravimetric analysis (TGA), X-ray diffraction (XRD)), and the extraction yields were calculated. Fine tuning the microwave treatment variables, it was possible to realize a simple, cost-effective way for faster materials’ preparation, which allowed achieving high-quality CNCs, with a defined hydrodynamic diameter (150 nm) and zeta potential (−0.040 V), comparable to those obtained using conventional heating, in only 90 min instead of 16 h.

## 1. Introduction

Cellulose-based materials have been used by our society for thousands of years for many daily-usage items. Nowadays, the most recent and innovative studies are oriented on this widespread natural polymer, in an attempt to increase the application of its properties, especially at the nano-scale level; the nanometric and crystalline forms of cellulose, known as cellulose nanocrystals (CNCs), have garnered a huge level of attention from the international scientific community and an exceptional appealing throughout the world that does not appear to be relenting. In particular, the food packaging industry, which is still heavily dependent on synthetic materials, is interested in the wider use of these new bio-nanoparticles, in order to increase the sustainability of its products and reduce their environmental impact. In our previous works, we have widely demonstrated that cellulose nanocrystals offer a barrier to gas diffusion comparable to that offered by synthetic barrier polymers employed nowadays (ethylene vinyl alcohol, polyvinyl alcohol, polyvinylidene chloride), even applying considerably lower coating thicknesses [1,2,3,4].

Actually, cellulose nanocrystals have long been regarded as a laboratory interest, evidenced by the significant rise in spin-offs and pilot-scale initiatives and in the number of patents published over the last few years on this theme [5]. At the time of the TAPPI Nanotechnology for Renewable Nanomaterials Conference (TAPPI Nano), in June 2014, there was only one commercial entity producing nanocellulose for market development: the Canadian CelluForce Co. Since then, the market has exploded and, today, only few years later, we can report that, worldwide, there are many commercial entities, producing nanocellulose at capacities beyond pilot plant scale (CelluForce, American Process Inc., Paperlogic, Borregaard). Beside these, numerous research facilities are producing nanocellulose, and several new lab and pilot plants have been announced; further, we believe there are numerous unreported lab scale facilities at universities, paper mills, and other sites. Furthermore, the global nanocellulose market is expected to register a very high CAGR (compound annual growth rate) during the forecast period, from 2018 to 2023 [6,7], so these emerging nanoparticles have the potential to play a major role in the 21st century in the development of advanced materials [8].

However, although nanocellulose applications have been studied since around 1980, only recently has its manufacturing become at least technically feasible [9]. To date, the production of nanocellulose at industrial scale is still restricted to limited number of companies with a narrow production (totaling ca. 6000 kg per day) [10]. Encouraged by the growing industrial interest, especially of the food packaging sector, for cellulose nanocrystals (CNCs), further research efforts and alternative methods have to be explored to make the industrial scale-up of nanocellulose production increasingly convenient and more simply achievable.

It is fair to assert that most of existing literature to date reports the typical procedure currently employed for the preparation of cellulose nanocrystals (CNCs) consisting of subjecting an appropriate cellulosic feedstock to a strong acid hydrolysis under strictly controlled conditions of temperature, agitation, and time. The most common protocol involves the use of mineral acids (typically sulfuric acid ca. 64% *w*/*w*) at the temperature range from 45 to 70 °C, for times varying according to the temperature [11,12,13,14]. These procedures are expensive, require considerably high initial capital investment, and have high operating costs owing to the corrosiveness, safety issues, and hazardous waste treatment/disposal requirements of such acids and their by-products. Additionally, they require relatively pure cellulosic starting materials, such as steam-exploded wood pulp and microcrystalline cellulose, or alkaline and bleaching agents, as pre-treatments to remove non-cellulosic fiber contents (e.g., lignin, pectin, hemicelluloses). The use of highly corrosive mineral acids, requiring expensive corrosion resistant equipment, multiple treatment steps, or tedious isolation techniques, impedes large-scale production and real-world applications of CNCs [15]. However, recent international scientific literature reported a simple and versatile one-step procedure to produce highly crystalline CNCs, which involves the use of ammonium persulfate ((NH_4_)_2_SO_8_), an oxidant with low long-term toxicity, high water solubility, and low cost. Recent studies have shown that the latter allows to obtain, in a single step, the hydrolytic fragmentation of cellulose, with the formation of nanocrystals, and the oxidation of some primary hydroxyl group into carboxylic ones, which is of great utility in the incorporation or grafting of CNCs in packaging materials and in the possibility of binding functionalizing molecules to CNCs [3]. This hydrolytic-oxidative process, also covered since 2014 by a patent (US 8,900,706) of Leung and co-workers [16], describes, for preparing cellulose nanocrystals, a heating treatment time that is substrate-dependent, ranging from 5 and 24 h for complex substrates and typically of 16 h for the most common cellulosic materials such as hemp, flax, and so on. The APS method is broadly applicable to a variety of native plant fibers and other cellulose sources, simultaneously removing lignin and amorphous cellulose to yield high-quality CNCs.

In the APS protocol, as for the ones with mineral acids, heating has traditionally been the conventional method, meaning that energy is conveyed through convection, conduction, and radiation. However, the rate of conventional heating is slow compared with microwave (MW) heating. In the latter way of heating, electromagnetic energy converts thermal energy through direct interaction of the incident radiation with molecules of a target material. Many experiments have shown that, under MW irradiation conditions, chemical reactions can be significantly accelerated by several orders of magnitude [17], and selectivity of the ensuing products can be obtained by choosing appropriate MW parameters, thus offering several advantages over conventional heating [18]. As a result, the microwave heating technique has emerged as a valuable alternative in the production of organic compounds, polymers, inorganic materials, and even nanomaterials, with shorter reaction time and higher reaction rate, selectivity, and yield compared with the conventional heating methods [17,19,20,21]. In recent years, some review articles have been published on the microwave irradiation-assisted synthesis of nanostructured materials, such as metal nanostructures [22], nanostructured carbon materials [23], nanoporous nanomaterials [24], colloidal nanocrystals [25], inorganic nanomaterials [26], metal oxide nanoparticles supported on carbon nanotubes [27], and polymer nanocomposites [28]. Some researchers have explored it for nanocellulose production [10,13,14] or modification processes including acetylation [29] and carboxymethylation [30], but no one commented on oxidative hydrolysis by means of microwave heating. Simple procedures, instantaneous and rapid heating, high temperature homogeneity, decreased energy costs, unique transformations, and ease of scalability are the major advantages that become apparent when using microwave-assisted chemistry. Moreover, as a result of the more efficient heating in the processes, microwave energy is understood to be more environmentally friendly, requiring less energy consumption than the conventional heating processes [31]. By controlling the specific MW parameters (temperature, pressure, and ramping of temperature) and choice of solvents, researchers can now move into the next generation of advanced nanomaterial design and development [32].

In contrast to conventional approaches, which are considered to be energy- and time-intensive, expensive, and not green, herein, we report an alternative microwave-assisted APS method for cellulose nanocrystals production, under pressurized conditions in a closed reaction system. The aim was to optimize the hydrolytic-oxidative procedure described by Leung et al.’s patent, replacing the conventional heating with a faster heating, based on microwave technology, which could allow the industrial scale production of the nanomaterials and make it more appealing to a green economy. In this paper, several features were evaluated in order to compare the nanocrystals raising from traditional and microwave processes and to investigate the influence of different thermal conditions on production and properties of nanocellulose materials (CNCs).

## 2. Materials and Methods

### 2.1. Materials

The cotton powder as raw material to be used for CNCs production was supplied by Sanitars S.p.A. (Flero, Brescia, Italy), who obtained it as waste product from cotton processing. The chemical reagents (ammonium persulfate ≥98%, sodium hydroxide ≥97%) were purchased from Sigma-Aldrich (Milan, Italy).

### 2.2. Design of Experiment

For the design of the experiment, a multilevel general factorial design was used, exploring two quantitative factors: the ramp time (*X*_1_), that is, the time required to reach a predefined heating temperature of 120 °C; and the hold time (*X*_2_), or time for temperature maintenance. On the basis of some previous experiences, four levels for the ramp time (5, 15, 30, 40 min) and two levels for the hold time (60, 90 min) have been set, with four replicates for each combination of experimental conditions. Moreover, four central points were employed in the design, for a total of 36 runs (runs), performed with a completely randomized blocks sequence. The effect of all the possible combinations of the levels of the two factors, *X*_1_ and *X*_2_, on some parameters of the nanocrystals were analysed: (I) hydrodynamic diameter (*D*_Hy_), (II) polydispersity index (*PDI*), (III) zeta potential (*ζ*), and (IV) yield (*Y*). The experiment design was supported by the Design-Expert software (version 7.0.0) of Stat-Ease^®^ (Stat-Ease Inc., Minneapolis, MN, USA).

### 2.3. Cellulose Nanocrystals Extraction Processes

#### 2.3.1. Heating Programs

Cellulose nanocrystals from the same raw material (i.e., cotton powder) were obtained using the two procedures as outlined next. For the conventional heating process, the milled cotton powder was subjected to an oxidative-hydrolysis with ammonium persulfate (APS) 1M solution according to the method of Leung and co-workers (Leung et al., 2011). The reaction mixture (ratio between fibers and APS of 10:1 g/L) was thus placed into a large beaker, onto a magnetic stirrer hotplate, equipped with a Vertex Digital thermoregulatory (VELP Scientifica, Usmate, Italy), heated at 75 °C, and continuously stirred for 16 h, limiting the evaporation by means of a plastic foil cover.

Instead, microwave-assisted preparation of CNCs involved that cellulosic material was introduced into microwave pressure vessels (HVT50, teflon, 50 mL) and 1M APS solution was added, maintaining the same rapport between fibers and reactive solution as the conventional heating method. The suspension was homogenised through stirring for 5 min and transferred to a laboratory microwave digestion system (Multiwave GO, Anton Paar, Graz, Austria) during the whole heating procedure. The equipment was provided with a single magnetron delivering up to 850 W microwave power over the full power range. For accurate reaction control, the vessel temperature was continuously monitored with an IR temperature sensor, measuring from the bottom of the cavity. The sensor system of the instrument enabled automatic detection of each reaction vessel, as well as monitoring of the rotor revolution, to ensure uniform microwave heating and prevent localized overheating. The microwave-assisted process was performed according to sequential heating steps, choosing different time-temperature programs; the reaction mixture has initially been ramped to a set temperature of 120 °C and then held to the fixed temperature before final cooling (till 40 °C). The rate of temperature increase (Δ*T*/*t*) remained constant according to the selected ramp time. The reaction conditions, for conventional and microwave heating, are summarized in Table 1.

**Figure 1:**
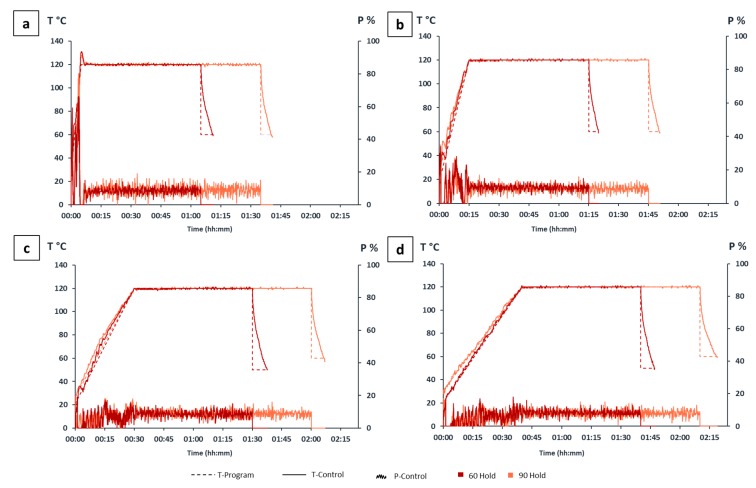
Monitoring of the temperature (°C) and microwave power supplied (%) during predefined heating programs: (**a**) M_1_, M_5_; (**b**) M_2_, M_6_; (**c**) M_3_, M_7_; (**d**) M_4_, M_8_.

The microwave reactor allowed precise control of reaction conditions and homogeneous temperature profiles, closely fitting with those programmed, as shown in Figure 1.

#### 2.3.2. Purification Steps

Aiming to concentrate the cellulose and remove excess acid, the suspensions of CNCs were subjected to subsequent centrifugation–washing procedures, at 4000 rpm for 20 min, using deionized water, until the pH level increased from around 0.2 to 4 (pH correction was then performed, increasing it to 8 ± 0.5 with NaOH to avoid aggregation of the crystals in an acidic environment). An ultrasonic treatment (UP200St 200W, Hielscher Co., Teltow, Germany) was then carried out, at 0.7 cycles of 20 min and 70% output, to distribute CNCs evenly in the suspensions. The suspensions were vacuum filtered using Munktell filter paper (grade 1–2 μm) to remove fibers that did not completely react with APS, and other big cellulose agglomerates and large contaminants that may have been introduced during the process. The purified suspensions were finally freeze-dried (LyoQuest -55/230V 50Hz, Telstar, Spain) for 3–4 days to achieve white CNCs powder.

### 2.4. CNCs Extraction Yield

The yield of CNCs production (%) was estimated, using a gravimetric method, by Equation (1), as the ratio of freeze-dried CNCs’ weight (*w*_2_, g) and the cellulosic content of raw material (*w*_1_, g), which is equal to 80.5% of the fresh weight of cotton powder:

Yield% = (*w*_2_/*w*_1_) × 100 (1)

The yield was calculated based on an average of four replicates for each heating condition.

### 2.5. Morphological Characterization of CNCs

The morphological characteristics of the CNCs were evaluated via transmission electron microscopy (TEM), using a LEO 912AB TEM with an Omega energy filter (Zeiss, Oberkochen, Germany) at an accelerating voltage of 120 kV. For the microscopic observations, drops of dilute aqueous suspension of CNCs (≈4 wt %), previously sonicated for 1 min, were deposited on carbon-coated electron microscope grids, negatively stained with 2% uranyl acetate, and allowed to dry. Representative micrographs were selected for measuring the diameter (D), length (L), and aspect ratio (L/D) of the nanocrystals by digital image analysis (iTEM, software, Olympus Soft Imaging Solutions GmbH, Münster, Germany). In addition, the dimensions of CNCs from all heating programs, in dilute suspensions (at pH 8), were also investigated by dynamic light scattering (DLS) measurements (mod. Litesizer500, Anton Paar, Graz, Austria), performed at 25.0 ± 0.1 °C with a 35 mW laser diode light (*λ* = 658 nm), and collecting the scattered light at 15° and 90°. By applying correlation analysis and the Stokes–Einstein relation, the equivalent hydrodynamic diameters (*D*_Hy_), the polydispersity index (*PDI*), and size distributions of the scatters were calculated. Four runs were performed, withdrawing three different aliquots for each set of experimental conditions.

### 2.6. Zeta Potential Measurement

The diluted suspensions of the CNCs (pH 8) were analysed by electrophoretic light scattering (ELS) (mod. Litesizer 500, Anton Paar, Graz, Austria), which allows to measure the electrophoretic mobility of the particles suspended in a liquid, directly proportional to their Zeta potential (*ζ*, mV), according to Henry’s equation. Measures were replicated four times, at 25.0 ± 0.1 °C, by means of a 35 mW diode laser (λ = 658 nm), and at a 15° detection angle.

### 2.7. Statistical Analysis

The two independent variables, ramp time (*X*_1_) and hold time (*X*_2_), were statistically explored, subjecting the data to a two-way analysis of variance (ANOVA) to determine the effects of the factorial combination of *X*_1_ (quantitative factor on four levels) and *X*_2_ (quantitative factor on two levels), each of which was repeated four times. The statistical significance of the model was determined by evaluating the *p*-value, F-value, and lack of fit at a 95% confidence level. The statistical treatment of the experimental data also consisted of fitting a polynomial function to the set of data collected from multilevel factorial design, by means of the least squares method. The extent of fitting was expressed by the adjusted determination coefficient (*R*^2^_adj_). Response surfaces were then obtained using the fitted model and keeping one independent variable constant while varying the other variable. Furthermore, to obtain the process settings achieving peak performance, a numerical optimization function was used to find the maximum desirability for all responses simultaneously, combining all the data into a single desirability function (D), through the geometric mean (D_j_) of the individual desirability.

Finally, to differentiate the CNCs samples of each series of microwave heating programs from those of conventional heating, the mean values of measured parameters were compared using Fisher’s least significance difference (LSD) test, where the F-test was significant and with a significance level of *p* ≤ 0.05. To this aim, a one-factor comparative experiment was performed, whose levels were represented by the different heating programs adopted (M_1_, M_2_, M_3_, …, C_1_).

### 2.8. Fourier Transform Infrared Spectroscopy (FTIR)-Attenuated Total Reflectance (ATR) Analysis of Cellulose Nanocrystals

The presence of CNCs was verified and further investigated using Fourier transform infrared spectroscopy (FTIR), which allowed to identify key chemical compounds through the chemical bonds’ absorbance of infrared radiation. The FTIR-ATR spectroscopy was performed, in duplicate, on the native cotton powder and on freeze-dried CNCs samples, resulting from conventional heating and from the microwave heating program, which provided the best results in terms of yield, size, and Zeta potential. A Perkin Elmer instrument (Spectrum 100) was used, equipped with attenuated total reflectance (ATR) accessory and a spherical Ge crystal, fixed at an incident angle of 45°. All spectra were collected at a spectrum resolution of 4 cm^−1^, with 180 scans and recorded, in transmittance mode, over the wavenumber range of 4000–600 cm^−1^. A background scan of clean Ge crystal was acquired before scanning the samples.

### 2.9. X-ray Diffraction (XRD) Characterization

X-ray diffraction (XRD) measurements were performed on the same freeze-dried powders that were analysed in FTIR-ATR, to gain insight into details of the crystalline structure of CNCs from the two heating methods. The diffraction patterns were detected, at room temperature, on a Panalytical X’pert PrO diffractometer (Malvern Panalytical S.r.l., Lissone, Italy) equipped with a copper (Cu-K_α_, *λ* = 1.5405 Å) rotating anode source, at an operating voltage of 40 kV and a filament current of 40 mA. The 2*θ* angles were recorded from 10° to 45°, with a step size of 0.02°, scanning rate of 2 s/step, divergence slit of 0.25°, soller slit of 0.04 rad, and antiscatter slit of 0.5°. The collected data were analyzed using Origin^®^ 2019b data analysis software to provide peaks position (2*θ*), full width at half maximum (FWHM), and peaks deconvolution. Diffraction peaks were profile-fitted assuming Gaussian–Lorentzian functions, as stated by the common experience [33,34,35,36]. Baseline anchor points, based on raw data, were automatically located through a second derivative baseline function and subsequently connected by interpolation. The interplanar distances in crystallites (*d*_hkl_-spacing) were calculated using the Bragg’s Equation (2):

*d*_hkl_ = *λ*/2 *sinθ* (2)

where *d*_hkl_ (nm) is the spacing between the planes in the atomic lattice, *λ* is the wavelength of X-ray radiation (nm), and *θ* is the angle between the incident ray and the scattering planes [35,37].

In addition, the X-ray diffraction patterns were used to determine the size *τ* (nm) of cellulose crystallites, through the Scherrer Equation (3):

*τ*_hkl_ = *kλ*/*β cosθ* (3)

where *τ*_hkl_ (nm) is the crystallite size perpendicular to the lattice plane, *K* is the Scherrer constant (0.94), and *β* is the FWHM of the diffraction peak in radians [38,39].

### 2.10. Thermogravimetric Analysis (TGA)

The thermal behaviour of the two kind of CNCs, from conventional and the best microwave program, was studied by employing a thermogravimetric analyzer Perkin Elmer, TGA 4000 (PerkinElmer Inc., Waltham, MA, USA) under an air and nitrogen flow, with a purge rate of 20 mL min^−1^. Samples, weighing from ca. 7 to 10 mg, were heated from 30 to 600 °C, at a heating rate of 10°C min^−1^. Two replications were done for each of the two types of CNCs. The differential thermogravimetric (DTG) curves were also obtained using differentials of TGA values by the following Equation (4):

*DTG* = (*w_T_*_+∆*T*_ − *w_T_*_−∆*T*_)/2∆*T* (4)

where *w_T_*_+Δ*T*_ and *w_T_*_−Δ*T*_ are the residual weights of the sample at temperature *T*+Δ*T* and *T*−Δ*T*, respectively, and Δ*T* is the interval of temperature for reading residual sample weight [40]. The onset and endset degradation temperatures of the samples were obtained from TGA curves, as the intersection of the tangents to the point of deviation from the initial and final weight, respectively, and the inflection point of the curve. To further define degradation, the temperature of maximum degradation rate (*Tv*_max_) was determined as the minimum point of the derivative curve, DTG, and thus corresponding to the inflection point of the TGA curve. The peak of the first derivative indicates the point of greatest rate of change on the weight loss curve.

## 3. Results

### 3.1. Evaluation of the Yield of Cellulose Nanocrystals

As shown in Table 2, the choice of heating program, with the other process conditions being equal, considerably affected the yield of CNCs, which is essential to decree the extraction success. It is worth noting that the reported yields are only indicative and related to a specific lab-scale production, as they strongly depend on the preparation procedure and post-treatment filtration.

The hydrolysis by conventional heating gave 48.85% ± 11.99% of cellulose nanocrystals, while the yields recorded for microwave-assisted hydrolysis were at most 45.81% ± 3.79%, when the ramp–hold combination was set according to M_3_ program, and tended to decrease when the ramp times progressively decreased, reaching just 22.09% ± 1.64% and 23.37% ± 2.98% for the shorter ones (i.e., 5 min, M_1_ and M_5_, respectively). On the other hand, no important differences between the two hold times were detected; the extraction efficiency of the process at 60 min hold time was, in fact, comparable to that obtained at 90 min, except for the yield of 30 min ramp processes, for which the procedure with the shorter hold time (60’) was significantly better.

Comparatively, the yield of conventional heating (C_1_) was very close to the highest for the microwave mode of heating (M_3_). Note that, in other previous works, APS treatment applied to the cotton raw material has led to CNCs with average yields that were comparable or even lower than that achieved using M_3_ method [40,41,42]. Additionally, the results were, in all MW heating programs, more repeatable, with standard deviations significantly lower than the conventional CNCs preparation procedure, thanks to the rigid control of the reaction conditions allowed by the microwave reactor.

### 3.2. Morphology of Cellulose Nanocrystals

**Figure 2:**
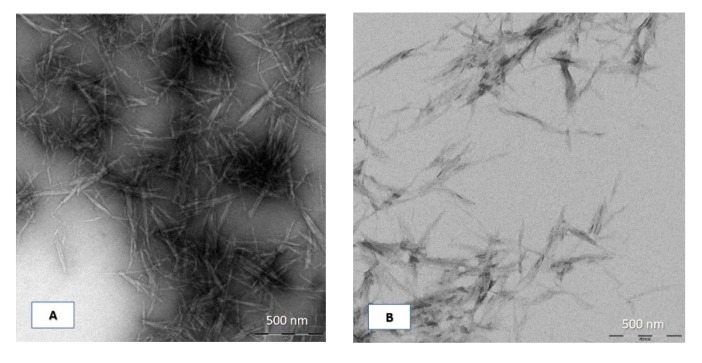
Transmission electron microscopy (TEM) micrographs of diluted suspensions of cellulose nanocrystals obtained by traditional (**A**) and microwave (MW) (**B**) heating methods.

The morphology and geometric dimensions of the nanocrystals (length, width, aspect ratio) strictly depend on the exact conditions in which the hydrolysis occurred, as well as on the cellulose source used [43]. On the basis of this consideration, a characterization of the actual shapes, particle dimensions, and length distributions of CNCs, via transmission electron microscopy (TEM) and by dynamic light scattering (DLS), was performed. The electronic micrographs (Figure 2A,B) revealed rather similar aggregates of the two sets of nanocrystals produced, with typical rod-like crystal structure and a rather high aspect ratio (greater than 20). The measured widths were approximately in the order of few nanometers (around 7 nm), for both kind of CNCs, while their length ranged over a larger window, from some tens (≈80 nm) to a few hundreds of nanometers (≈400 nm), with an average value of around 170 nm. However, it is known from the literature that CNCs generally have a relatively wide length distribution, owing to the controlled nature of the progress of the hydrolytic process [44]. Moreover, the dimensions of the cellulose nanocrystals were comparable to those from previously reported works [8].

The average CNCs’ lengths achieved from TEM observations closely matched the DLS mean values of hydrodynamic diameter (Table 2). It is worth highlighting that the average D_Hy_ tended to be lower as the ramp time increased, varying from 212 ± 14 nm and 244 ± 26 nm of M_1_ and M_5_, to 165 ± 6 and 159 ± 8 nm of M_4_ and M_8_, respectively. However, by setting the heating program M_3_, the smallest D_Hy_, 153 ± 5 nm, was reached, which increased from M_3_ to M_4_ or by prolonging the hold time to 90′.

The CNCs’ dilute dispersions exhibited a bimodal frequency length distribution, which mainly ranged (*α* = 0.1) from 91 nm to around 330 nm, except for M_1_, from 116 to 498 nm, and for M_5_, from 126 to 460 nm (data not shown); these were comparable to the range of lengths obtained using the electronic microscope, still indicating the good overlap between size distribution in CNCs solution and electron micrographs of drop-casted CNCs. Polydispersity index (*PDI*) is a measure of the width of particle size distribution. A value below or equal to 10% refers that the sample is monodisperse [2,45]. The higher polydispersity indexes of the CNCs obtained (Table 2) were thus indicative of a more complex morphology and bi- or multi-modal distributions.

### 3.3. Zeta Potential Analysis

For each heating method, the average Zeta potential of diluted suspensions of CNCs is shown in Table 2. Except for M_1_ and M_5_, with Zeta potentials (*ζ*) of −27.39 ± 3.28 mV and −26.56 ± 1.17 mV, respectively, the conventional heating and the remaining microwave programs recorded a voltage value lower than −30 mV, which reflects cellulose nanocrystals stably dispersed in the colloidal suspension [46]. In particular, the combination of process factors (ramp time, hold time) defined in the M_3_ program produced crystals with the highest Zeta potential (−40.57 ± 2.53 mV), comparable to those of the nanoparticles resulting from C_1_. The progressive increase (in absolute value) of the Zeta potentials with the increase of the ramp time should be noted, while no important change was found with the changing of the hold time. The only exception, once again, is M_3_ program, which emerged with higher potentials than M_7_, having a longer hold time (90 min), but the same ramp time (30 min).

### 3.4. Statistical Evaluation of the Responses and Model Fitting

The statistical processing of collected data was carried out using a quadratic model, considering the superior abilities to predict and explain the variability of the data compared with the linear model (it ensured the highest R^2^_Adj_). As shown in the Table 2, for hydrodynamic diameter, Zeta potential, and yield responses, analysis of variance (ANOVA) confirmed the adequacy of the second-order model, with the model probability value (*p*-value) falling below 0.001, and exhibiting a not significant lack of fit (*p*-value > 0.05). For these responses, the model showed a good fit with the experimental data, as the values of the adjusted determination coefficient (*R*^2^_adj_) were quite high, at 0.8 for *D*_Hy_, 0.7 for *ζ*, and 0.8 for *Y*.

Both factors studied, *X*_1_ (beside its quadratic term *X*_1_^2^) and *X*_2_, showed significant main effects for the hydrodynamic diameter, while the interaction between the two (*X*_1_*X*_2_) showed a *p*-value greater than 5% (*p* > 0.05), which made it not significant for the same responses. However, the F-value for *X*_2_ was very small compared with the F-value of *X*_1_ (data not shown), and this implied that the latter factor had a much larger effect on the response variable. Instead, for the Zeta potential and yield responses, only *X*_1_ and *X*_1_^2^ were significant for ANOVA. Moreover, it should be noted that neither of the two factors examined (ramp and hold time), nor their interaction, were significant as far as the polydispersity index (PDI) is concerned. 

In order to know the effect of non-experimental intermediate levels, the interpolation equations of the response variables were calculated, quantitatively describing the behaviour of the system. The final equations of the regression predictive models are reported below, in terms of coded factors (Equations (5)–(7)):

(*D*_Hy_)^−1.8^ = + 30.08 + 6.92 *X*_1_ − 1.40 *X*_2_ − 5.93 *X*_1_^2^ (5)

*ζ* = −0.034 − 0.0047 *X*_1_ + 0.0034 *X*_1_^2^ (6)

*Y* = 36.60 + 6.91 *X*_1_ + 8.18 *X*_1_^2^ (7)

**Figure 3:**
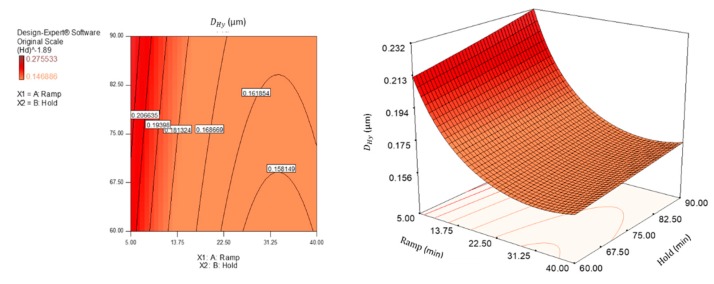
Response surface (**right**) and contour plot (**left**) showing the effect of ramp (*X*_1_) and hold (*X*_2_) on the response variable hydrodynamic diameter (*D*_Hy_).

**Figure 4:**
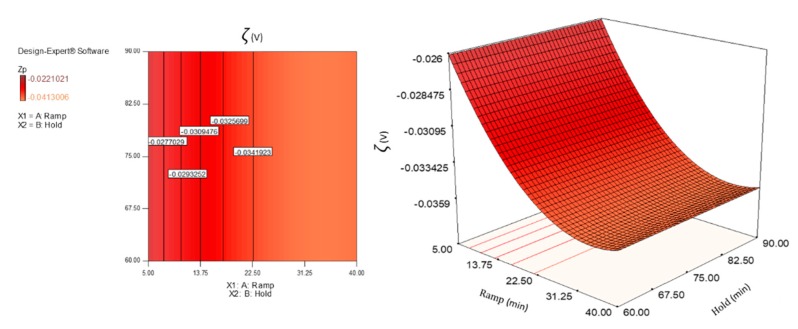
Response surface (**right**) and contour plot (**left**) showing the effect of ramp (*X*_1_) and hold (*X*_2_) on the response variable Zeta potential (*ζ*)

**Figure 5:**
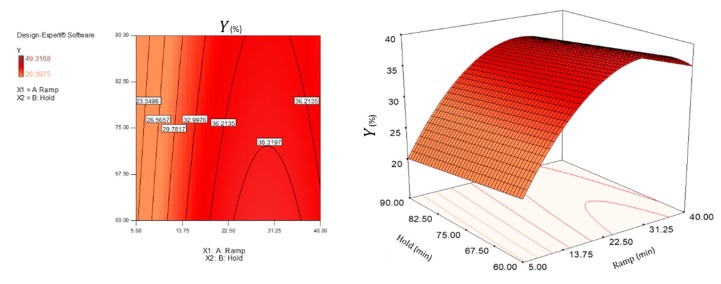
Response surface (**right**) and contour plot (**left**) showing the effect of ramp (*X*_1_) and hold (*X*_2_) on the response variable Zeta potential (*Y*).

**Figure 6:**
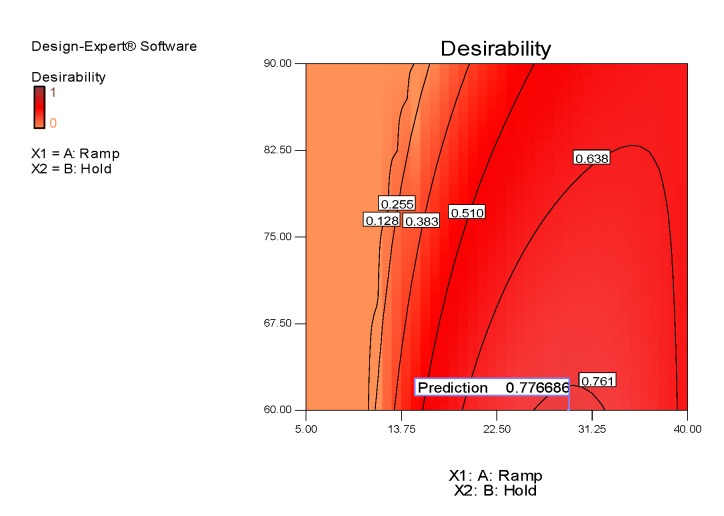
Contour plot of desirability function (desirability plot) showing the prediction values satisfying the restriction criteria in the desirability area.

By default, in the equation in terms of coded factors, the high levels of the factors are coded as +1 and the low levels as −1. The equations show the factor coefficients, calculated using the least square technique, each of which represents the expected change in the response per unit of variation of the independent variable, when all the remaining factors are kept constant. The intercept is the overall average response of all executions. For the hydrodynamic diameter response, the power transformation was applied, using the Box Cox diagram of Design Expert, in order to improve the matching of the model to the data. Three-dimensional surfaces and contour plots were generated based on Equations (5)–(7), and are shown in Figure 3, Figure 4, Figure 5 and Figure 6. In the graphs, the model with quadratic terms highlighted the presence of curvature, but no marked torsions were found, owing to the not significant interaction of the two factors for the analysis of variance.

With the aim of optimizing the responses, criteria and target values maximizing the desirability function (d_j_) were assigned. It was assumed that was necessary to minimize the hydrodynamic diameter, maximize (in absolute value) the Zeta potential setting the lowest acceptable threshold to −30 mV, and maximize the extraction yield. Adopting the constrained optimization algorithm [47], the contour plot of the desirability function was constructed, with all the equivalent solutions able to satisfy the restriction criteria set, finding a maximum d_j_ of 0.77 (d_j_ ranges from zero to one for any given response) by combining a ramp time of about 29 min and a hold time of 60 min.

Finally, aiming to further explore the differences of the two heating systems, all the possible pairwise comparisons between the mean of each one MW program with the mean of C_1_ (conventional) were analysed, following one-way analysis of variance and using Fisher’s least significant difference (LSD) test, given that the null hypothesis had already been rejected. The results of LSD test confirmed the significant differences existing among heating programs in terms of yield, particle size, and Zeta potential. However, as shown in Table 2, for yield and Zeta potential responses, no significant differences between C_1_ and M_3_ emerged, as well as for M_2_, M_6_, and M_7_ methods with regard to the hydrodynamic diameter.

### 3.5. FTIR-ATR Analysis of Cellulose Nanocrystals

**Figure 7:**
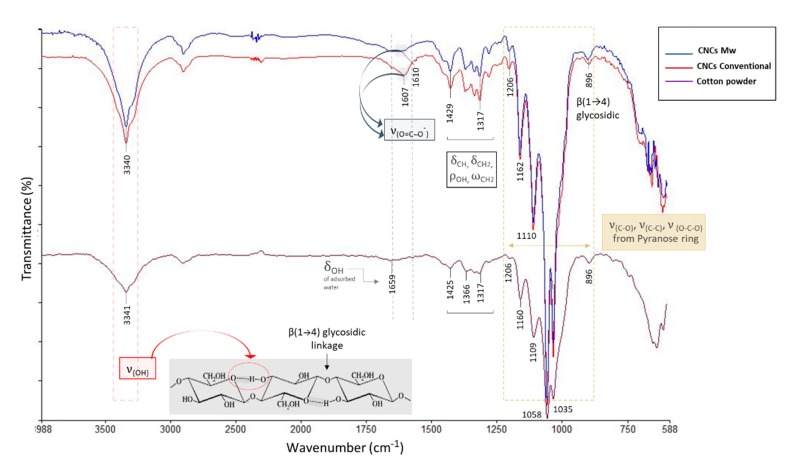
Fourier transform infrared spectroscopy (FTIR)-attenuated total reflectance (ATR) spectra (4000–600 cm^−1^) of cellulose nanocrystals (CNCs) from microwave (–––blue line) and conventional (–––red line) methods in comparison with the raw cotton powder (–––purple line).

By observing the changes occurring in the initial chemical structure owing to APS treatment, the characterization of the crystals by infrared analysis led to the double result of verifying the positive outcome of cellulose nanocrystals extraction process, as well as comparing the nanocrystals obtained with the two heating methods. FTIR-ATR spectra, of both type of CNCs produced, showed absorption bands surely typical of cellulosic materials and consistent with others’ reports. They revealed similarities, in transmittance, in the main regions highlighted in Figure 7.

The broad band centred at around 3340 cm^−1^, in all spectra, is attributed to the intramolecular hydrogen bonded O–H stretching vibration (ν_OH_) [48], in particular, that formed between O(3)H–O(5) positions, adjacent to the β-glycosidic bond of cellulose I [16]. It is known that the hydroxyls, linked to C(2), C(3), and C(6) carbons of cellulose, contribute to the formation of various types of inter- and intra-molecular hydrogen bonds, whose presence not only has a strong influence on the physical properties of cellulose, such as solubility, hydroxyl reactivity, and crystallinity, but also plays an important role in the mechanical properties of the polymer [49]. It is possible to observe that the amplitude relative to this absorption peak could be affected in the chain cleavage process, owing to the weakening of the hydrogen bonds throughout the hydrolysis [50]. In the spectra of CNCs, unlike raw materials, this band becomes, in fact, narrower and longer. Next to this region, FTIR peaks at 2900 cm^−1^, weak and diffused, are the result of –CH_2_ and C–H stretching vibrations in the cellulose structure [51]. The lower range of cellulose IR spectra, from 1800–600 cm^−1^, had relatively well-defined peaks. It is certainly worth highlighting the presence of absorption peaks at around 1600–1610 cm^−1^, probably attributable to carboxylate asymmetric stretching (ν_COO−_), which is formed because of primary hydroxyl groups’ oxidation, induced by APS treatment. In the conventional treatment, this band emerges as slightly stronger than the microwave one. However, it should be noted that the identification of the carbonate ion band around 1700–1600 cm^−1^ by FTIR-ATR is quite difficult because the O–H bending (δ_OH_) of absorbed water (1633–1659 cm^−1^) was also observed in this region [52,53]. FTIR data also suggested that oxidation occurred preferentially at the C6 primary alcohol of crystalline cellulose. The oxidation of C2 and C3 secondary alcohols is known to induce the cleavage of the glucopyranose ring, resulting in harmful lowering of crystallinity [54]. The absence of an IR signal relating to hemiacetal formation (880 cm^−1^) confirmed the intact crystalline structure of CNCs prepared by APS [15] with two adopted heating methods.

Cellulose molecules also contain several C–C and C–H bonds, which require numerous absorptions throughout the spectral range, but especially in the fingerprint region. The presence of signals at 1420–1430 cm^−1^ is assigned to the –CH_2_ scissoring in cellulose [55], as well as the 1317 cm^−1^ relative to the –CH_2_ wagging deformation mode (*ω*_CH2_) at C6, and 1281 cm^-1^ to the C–H bending. The medium weak peaks, around 1370 and 1334 cm^−1^, are attributed, instead, to OH out-of-plane bending vibrations [56,57] and to the rocking of –OH (*ρ*_OH_), respectively. The peak at 1110 cm^−1^, present in all the spectra, is attributed to the asymmetric stretching of the C–O–C group of cellulose. Other characteristic absorption peaks, related to the chemical structure of cellulose, were 896 cm^−1^, resulting from the β-glycosidic linkage [52], and 1058 (the strongest band across the cellulose spectra) and 1035 cm^-1^, referable to the –C–O and C–O–C bonds of the pyranose ring, respectively. It can also be observed that the presence of these very strong absorption bands, referable to the glucopyranose unit vibrations, demonstrates that the degradation of cellulose could hardly have taken place at the glucose rings. The main degradation point should, in fact, be the glycosidic bonds [50]. Absorption patterns of cellulose remained unchanged after persulphate treatment; this confirms that there are no significant changes related to the conformation of the cellulosic structure. With regard to the glycosidic bond, it is to be noted that, although the C1–O–C4 bond is highlighted in the FTIR analysis, the structure of cellulose molecules does not easily allow those vibrational movements such that the infrared ray is able to trigger and the FTIR is able to detect. For this reason and because of the relatively small number of broken glycosidic bonds (compared with the thousands present), the FTIR technique is not suitable to signal a decrease in the degree of polymerization following hydrolytic cleavage. Therefore, in the spectra of both CNCs, the peak at around 896 cm^−1^ remained substantially identical with respect to the cotton powder raw material.

In general, the comparison of these spectral data revealed that all CNCs are composed of crystalline cellulose I [16,55], with minimal differences in some peaks, while the absorption peaks around 1338, 1507, and 1734 cm^−1^, typically referable to the aliphatic carboxylic groups, aryl ester, and acetyl groups in the xylan, respectively, because of the presence of characteristic groups of hemicellulose and lignin, were totally missing. The reduction in the intensity of some peaks, in the infrared spectrum of the raw material (cotton powder), may be because of the presence of a network of bonds (hydrogen, glycosidic) that prevented or reduced some flexing and out of plane movements, which instead appeared in the crystalline structure of the CNCs.

### 3.6. X-ray Powder Diffraction Analysis

**Figure 8:**
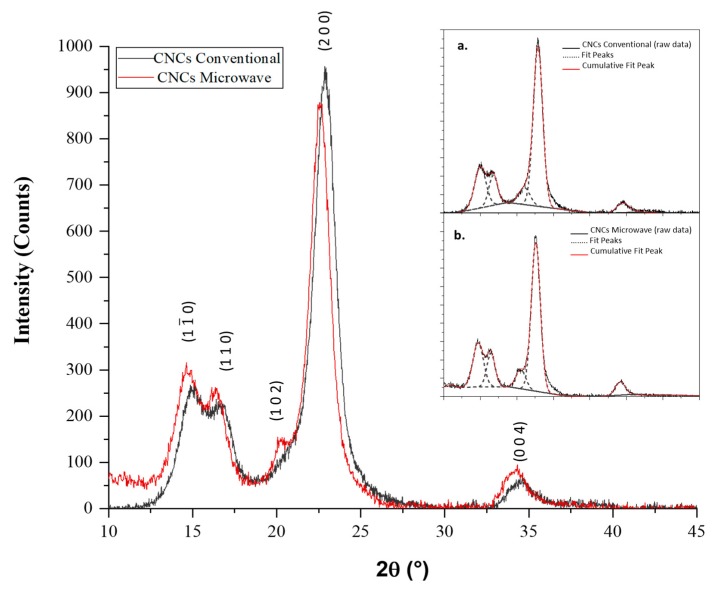
Experimental X-ray diffractograms for cellulose nanocrystals from conventional (thick black line) and microwave (thick red line) heating methods. In the insets, the fitted crystalline peaks (dashed grey line) and total fitted patterns (thin red line) for conventional (**a**) and microwave (**b**) CNCs are shown.

From the XRD characterization of CNCs, a few considerations were made on the type of crystalline allomorph and sizes of crystallites, and the integrity of the crystalline structure of both types of CNCs was confirmed. Four characteristic peaks, at around 15°, 16.5°, 22.8°, and 34.5° 2θ, were deconvoluted from the background scattering by the curve-fitting process (deconvoluted peaks are plotted in Figure 8 insets), corresponding to the (1 $\bar{1}$ 0), (1 1 0), (2 0 0), and (0 0 4) crystallographic planes, respectively [58,59,60]. Table 3 provides a comparison of the crystallite sizes and *d*-spacing values of the two types of CNCs, evaluated along the three main planes (1 $\bar{1}$ 0), (1 1 0), and (2 0 0), which were consistent with the most references [37,61,62,63].

Such features, including the *d*-spacing and average crystallite size, as determined by the Bragg’s and Scherrer’s equations, resembled the diffraction pattern of cellulose I_β_, according to crystallographic data reported in our previous work [3]. The peaks discussed in the following crystalline analysis also indicated that crystal structures of CNCs remained unchanged during APS oxidative hydrolysis, with both conventional and microwave heating. 

Diffractograms also exhibited a shoulder on the (2 0 0) peak, at around 20.5 2*θ* angles, which was ascribed to the (1 0 2) crystallographic plane of cellulose I_β_ phase [59]. Comparing the diffraction patterns of the two samples, this peak was more discernible and intense for MW CNCs than for the conventional one. This is a characteristic for crystals with the Miller index (1 0 2) in cellulose type I polymorph, which does not always appear in all type I cellulose samples [34,39]. Five crystalline peaks (1 $\bar{1}$ 0, 1 1 0, 1 0 2, 2 0 0, and 0 0 4) were separated in many cases [64], but four crystalline peaks (1 $\bar{1}$ 0, 1 1 0, 2 0 0, and 0 0 4) were assumed in other studies [40,65].

### 3.7. Thermal Properties of Cellulose Nanocrystals

**Figure 9:**
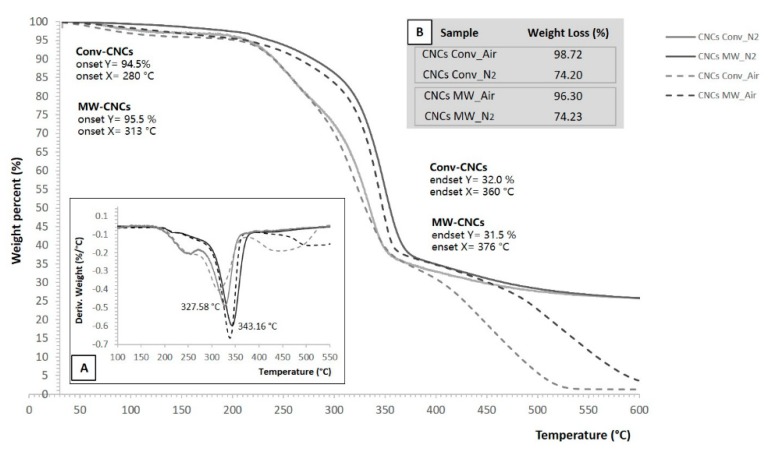
Thermogravimetric analysis (TGA) and differential thermogravimetric (DTG) curves (inset **A**) of CNCs samples in air and nitrogen atmosphere. Final weight losses (%) of CNCs in both atmospheres (inset **B**).

Thermal stability was also tested given its importance in highlighting the potential differences between the two nanocrystals. Furthermore, it plays a critical role in the preparation of melt processed CNCs composites for thermoplastic applications [66]. The thermogravimetric (TGA) and differential thermogravimetric (DTG) profiles of the conventional CNCs and those of the MW treated CNCs are shown in Figure 9.

Leaving aside the temperature range between 30 and 200 °C, where the differences of the two types of CNCs appear to be negligible, with ˂5% mass loss below this temperature, TGA curves of the MW nanocrystals exhibited different thermal behaviours compared with those conventionally produced. This suggested that, after the weight loss related to the moisture removal, the chemical characteristics of CNCs played a role in weight loss. From 200 °C, a sharp degradation occurred until the major break-up appeared at a temperature over 400 °C, owing to the complete decay of CNCs to volatile products. In particular, two main degradation events characterize the mass loss profile of nanocrystals under N_2_ atmosphere; that is, thermal decomposition at onset temperature above 250 °C until nearly 340 °C, where CNC underwent their first important degradation loss from 15% to 30% of their mass (Td_15_, Td_30_), followed by another 30% mass loss in the 340–370 °C region (Td_60_). These phenomena could be attributed to the progressive thermal depolymerization of the polymeric chains to 1,6 anhydro-β-d-glucopyranose which starts to gasify efficiently above 300 °C [67]. At temperatures higher than 400 °C, there is oxidation and breakdown of the charred residue to lower molecular weight gaseous products and CO_2_ [68].

Interestingly, in comparison with conventional nanocrystal samples, microwave CNCs exhibited higher thermal stability for all phases of thermal decomposition. The onset degradation temperature (Td_onset_), obtained from TGA curves, was a few tens of degrees lower for conventional nanocrystals (event shown in Figure 9). It could be appreciably observed, from the thermograms, that increments of 47.82 (ΔTd_15_) and 29.78 °C (ΔTd_30_) occur in the MW CNCs at the temperature corresponding to the 15% and 30% weight loss, respectively. Likewise, the value of Td_60_ for conventional nanocrystals weakly decreased to about 347.62 °C, compared with 367.19 °C of MW crystals (ΔTd_60_ = 19.57 °C). The endset (Td_endset_) and the temperature of maximum degradation rate (T*v*_max_) (deduced by peak calculation of the first derivative of the weight loss curve), in N_2_ atmosphere, were also shifted to a higher temperature, from 360 to 375 °C and from 327.58 to 343.16 °C, respectively, in the case of MW CNCs. The difference in thermal degradation behaviour could be justified by the presence of a higher carboxyl content on the conventional CNCs surface, which is supported by the FT-IR spectra. Finally, heating in air induced a significant decline in the decomposition profile, leaving significantly lower final residues, nearly 4% and 1.3% (Figure 9 inset B), than those of nitrogen atmosphere, around 26%.

## 4. Discussion

The APS oxidative-hydrolysis, performed with both heating modes, was effective in reducing the particle dimensions of native cellulose while allowing satisfactory yields, although rather variable according to the different heating programs. In particular, too rapid achievement of high temperatures (i.e., the use of shorter ramp) has probably depleted part of the reagent used to extract and oxidize the CNCs, before free radical ions, generated by persulfate, could actually penetrate through the lumen of the fibers and perform the shortening of cellulose into crystallites. This left an important fraction of the polymer undissolved and led to lower yields. Furthermore, during the heating run, the generation of a lot of energy and heat (self-heating) was observed, attributable to the triggering of spontaneous and exothermic reactions. The exothermic reactions can be recognized in Figure 1 by an increase of temperature while the microwave power is reduced or even shut off. At that point, significant amounts of reaction gases and heat are generated by the decomposition of the sample. As result, the microwave power input is reduced to keep the temperature value at or below the set limit. However, using the short ramp methods, the offsetting of this phenomenon with the drastic power drop was not enough and, therefore, temperature spikes were generated (overshoot), failing the set temperature (Figure 1a,b). Moreover, the high-temperature hydrolysis was accompanied by darkening of nanoparticles due to the intensification of oxidation, dehydration, and carbonization, which was also accompanied by caramelization of dissolved by-products of cellulose hydrolysis such as oligosaccharides [69]. Conversely, a slow ramp (≈30 min) enabled an appropriate heating rate for the type of sample that had to be digested. This made sure that no overloading and/or overheating of the vessels occurred, maintaining a reliable control of the process; using a slow ramp, all sample temperatures were equal to the target temperature and the exothermic reactions had enough time to happen at low reaction temperatures.

Another consideration is that, for the 30 min ramp processes, the yield achieved with the shorter hold time (60’) was significantly better than that obtained when prolonging the hold time up to 90’. This was ascribed to the creation of irreversible tight connections between the individual crystals released, owing to the extension of the heating time, and to the subsequent loss of these larger particles, during the purification steps (filtration above all). About that, it has been reported that the crystallites can grow in size, because of the large freedom of motion, after hydrolytic cleavage [8]. Taking this result into account, it could be considered more advantageous, in terms of saving energy and time, to adopt thermal programs with a shorter hold time.

It should also be noted that M_3_ heating program allowed extraction yield comparable to that obtained from conventional heating in a much longer reaction time. This may reflect the relative efficiency and specificity of microwaves in heating raw material, as it needs a very short time, only 90 min, instead of 16 h required by conventional one, leading to energy saving and high convenience in CNCs production. The treatment of raw material has in fact generated ionic species, derived from the thermal decomposition of persulfate in water solution [3], creating an in situ catalytic environment that proved to be advantageous under microwave irradiation [17], in hydrolysing hemicellulose, amorphous cellulose, and other components.

The results obtained through digital micrographs (TEM) were in a good agreement with those of dynamic light scattering (DLS), thus the latter appeared to be a rapid method sufficiently accurate to estimate the sizes of CNCs. In this regard, it should be noted that the DLS technique analyzes the data in spherical approximation, mathematically processing the CNCs as spheres that move with Brownian motion, regardless of their real physical morphology. The hydrodynamic diameter (D_Hy_) of a non-spherical particle thus corresponds to the diameter of a sphere that has the same translational diffusion speed as the particle. If the shape of a particle changes in a way that affects the diffusion speed, then the hydrodynamic size will change. For example, small variations in the length of a rod-shaped particle will directly affect the size, while changes in the rod’s diameter (cross section of the particle), which will hardly affect the diffusion speed, will be difficult to detect. In the case of CNCs, the average lengths achieved from TEM observations closely matched the DLS mean values of hydrodynamic diameter. These values tended to be lower as the ramp time increased, but reached the smallest value using M_3_ heating program. Moreover, *D*_Hy_ increased from M_3_ to M_4_ or by prolonging the hold time to 90′. This confirmed the hypothesis of an irreversible rearrangement of the crystals that could also explain the reduction in yield.

Further chemical feature was evaluated to compare the CNCs obtained from the two different heating methods. By means of light scattering, besides the hydrodynamic diameter, the Zeta potential (*ζ*) of CNCs was assessed using the electrophoretic light scattering (ELS) technique. Zeta potential value is an assessment of the electro-kinetic potential in colloid dispersions, being related to the degree of repulsion between ions of same charges and to the particle–particle interaction. It provides information about the particles’ surface-solvent interface, and its absolute magnitude is critical to understand the suspension stability and aggregation phenomena; therefore, it is fundamental to know it for the potential use of CNCs as coatings for flexible packaging materials. Not all kinds of CNCs produced exceed −30 mV, assumed as essential to guarantee favourable stability of colloid dispersions for an extended time (*ζ* < −30 mV or *ζ* > +30mV) [46]. In particular, the average Zeta potential of nanocrystals was determined to be below this threshold (in absolute value) only for the two microwave programs that involved the shortest ramp times, M_1_ and M_5_, respectively.

Interpreting the response surfaces generated by the final equations of regression predictive models, it could be concluded that, within the experimental domain, smaller nanocrystals with a higher Zeta potential and greater yield would be obtained by adjusting the process conditions to a high ramp level (i.e., by choosing longer ramp time) and at a low hold level (i.e., by choosing shorter hold time). However, the ramp factor (and its quadratic term producing the curvature) contributed greatly to influencing the answers, while only the hydrodynamic diameter was slightly affected by the hold time (*X*_2_), much less significant than the first factor (highly significant).

Through the contour plot of the desirability function, prediction values really close to the M_3_ method were found to be the optimal reaction conditions to achieve peak performances. Under these conditions, the best reaction environment for the isolation of CNCs could occur, that is, greater availability of the acid molecules at higher concentrations and enough time available for the simultaneous hydrolysis and oxidation process of cellulose fibers. Otherwise, the optimal potential of the reaction would decrease, owing to the premature degradation of the reagent and the triggering of carbonization reactions.

The cellulose I_β_ crystalline structure remained unchanged during APS oxidative hydrolysis with both conventional and microwave heating, as shown in the XRD measurements. The CNCs crystalline planes can be ordered in a different way depending on the synthesis conditions; a potential variation between the distances of the crystalline planes could be related to the stress and deformation present within the CNCs’ crystallites, and finally having an effect on the physical properties at macroscopic level. It has been previously reported that this affects the barrier behaviour of CNCs in permeability and migration phenomena, as well as their mechanical properties, that are very important for the future packaging materials that can be developed using these CNCs [39,70]. Therefore, the similar patterns and intensity preservation of diffraction peaks, after replacement of conventional heating with microwave heating, implied that the latter has not destroyed or converted the inherent crystalline structure of nanocrystals, which contributes to validating the designed MW method (M_3_) for CNCs production from cotton powder.

The thermogravimetric patterns confirmed the higher thermal stability of microwave CNCs for all phases of the thermal decomposition. Indeed, the higher carboxyl content on the conventional CNCs surface, in comparison with MW CNCs, as it appeared in the FTIR spectra, might result in worse thermal stability. This would be in good accordance with the results reported by others [3,60,71]. In addition, by determining the TG patterns in air, it seemed that more carboxylate groups on the latter nanocrystals could be introduced, in such an oxidizing atmosphere, inducing a significant decline in the decomposition profile, and leaving significantly lower final residues than those of nitrogen atmosphere. Heating in air causes, in fact, oxidation of the hydroxyl groups, resulting, as the temperature increases, in increases of carbonyl, carboxyl, and hydroperoxide groups, with free radicals also appearing. The thermal degradation in this case is accelerated [8].

## 5. Conclusions

Compared with conventional heating, the MW-assisted APS method has been proven to be an advanced technology in reducing the hydrolysis time of cellulosic amorphous regions, as it needs a shorter time, of only 90 min, instead of 16 h required by conventional heating, leading to energy saving and high efficiency in CNCs production. Fine tuning the treatment variables (ramp and hold heating time), we developed a simple, cost-effective way for a rapid and reproducible preparation of the nanocrystals, using cotton powder as raw material.

At optimal reaction conditions (M_3_ method), CNCs with a narrow size distribution, average particle length of 153 nm (hydrodynamic diameter), and average particle width of 7 nm were prepared in 45% of yield, comparable to those obtained by conventional method in a much longer reaction time. The nanoparticles formed stable suspensions in water with an average Zeta potential of −0.040 V, as measured by ELS. The resulting products retained the cellulose I_β_ crystalline structure as shown in the XRD measurements, and the FTIR spectral data were in perfect agreement with the data of the conventional method. Of further importance to compare the two types of nanoparticles, were the thermogravimetric patterns, confirming the higher thermal stability of microwave CNCs for all phases of the thermal decomposition.

Considering the environmental, chemical, and economic advantages introduced by this novel approach, microwave-assisted technology combined with other green chemistry strategies such us the use of APS could make the CNCs production more interesting from an economical and industrial point of view, as well as more appealing to a green economy, which should be greatly advocated and encouraged as a promising research trend.

The uniformity; small diameter; and high values of aspect ratio, Zeta potential, crystallinity, and thermal stability of such nanocrystals, which are assured by the good process control of the microwave reactor, allow to hypothesize their real applications as nanofillers or nanocoating in the food packaging field. However, in order to better characterize the properties of CNCs and their performances once they have been coated or introduced into food packaging materials, further investigations will be necessary in order to verify the analogy of CNCs resulting from the two heating methods. Moreover, the microwave-assisted technology could pave the way for observation of an increase in the carboxylation degree of CNCs by combining the oxidizing action of APS with the addition of an auxiliary oxidizing gaseous agent.

## 6. Patents

For the hydrolytic-oxidative microwave-assisted CNCs production described herein, an Italian patent was deposited as industrial invention (n° 102018000007870) and, more recently (2019.08.02), a European extension has been submitted (n° EP 19000360).

## Figures and Tables

**Table 1:** Microwave and conventional heating programs for the production of cellulose nanocrystals (CNCs).

**Microwave Heating Method**	**Heating Program**				**Total Heating Time (hh:mm)**	**Cooling** **(°C)**
	**Ramp (mm:ss)**	**Δ*T*/*t* (°C min^−1^)**	**Hold (mm:ss)**	**Temperature (°C)**		
M_1_	05:00	20	60:00	120	01:05	40
M_2_	15:00	7	60:00	120	01:15	40
M_3_	30:00	3	60:00	120	01:30	40
M_4_	40:00	2.5	60:00	120	01:40	40
M_5_	05:00	20	90:00	120	01:35	40
M_6_	15:00	7	90:00	120	01:45	40
M_7_	30:00	3	90:00	120	02:00	40
M_8_	40:00	2.5	90:00	120	02:10	40
**Conventional Heating Method**				**Temperature (°C)**	**Total Heating Time (hh:mm)**	**Cooling** **(°C)**
C_1_				75	16:00	40

**Table 2:** Mean values ±SD of experimental parameters for CNCs produced by conventional and different microwave heating programs. Analysis of variance (ANOVA) below.

	Heating Method	Yield *Y* (%)	Particle Size *D*_Hy_ (μm)	Polidispersity Index (*PDI*) (%)	Zeta Potential *ζ* (mV)
**Microwave**	M_1_	22.093 ± 1.634	0.212 ± 0.014	23.491 ± 0.926	−27.388 ± 3.280
	M_2_	32.314 ± 1.474	0.174 ± 0.008 ^a^	23.550 ± 1.637	−30.520 ± 1.638
	M_3_	45.814 ± 3.795 ^a^	0.153 ± 0.005	21.901 ± 1.172	−40.575 ± 2.534 ^a^
	M_4_	36.894 ± 3.236	0.165 ± 0.006	22.996 ± 2.649	−35.735 ± 2.077
	M_5_	23.370 ± 4.124	0.244 ± 0.026	22.760 ± 2.215	−26.559 ± 1.171
	M_6_	32.422 ± 2.985	0.178 ± 0.005 ^a^	21.842 ± 2.475	−32.095 ±2.466
	M_7_	34.411 ± 2.011	0.174 ± 0.009 ^a^	24.418 ± 2.999	−33.229 ± 2.844
	M_8_	35.031 ± 1.935	0.159 ± 0.008	22.714 ± 1.783	−35.874 ± 2.127
**Conventional**	C_1_	48.850 ± 11.991 ^a^	0.176 ± 0.002 ^a^	25.067 ± 0.231	−39.52 ± 1.141 ^a^
	Model	***	***	NS	***
	Lack of Fit	NS	NS	NS	NS
	Main effect				
	Ramp time (*X*_1_)	***	***	NS	***
	Hold time (*X*_2_)	*	NS	NS	NS
	Ramp time^2^ (*X*_1_^2^)	***	***	NS	**
	Interaction				
	Ramp × Hold (*X*_1_*X*_2_)	NS	NS	NS	NS
	Ramp^2^ × Hold (*X*_1_^2^*X*_2_)	NS	NS	NS	NS
	Performance index of fitted model				
	*R* ^2^_Adj_	0.8	0.8	-	0.7

***, **, and * indicate significant at *p* ≤ 0.001, *p* ≤ 0.01, and *p* ≤ 0.05. NS, not significant. ^a^ Same letter indicates statistical differences smaller than least significant difference (LSD) (not significant for *p* ≤ 0.05).

**Table 3:** Crystallographic parameters for conventional and microwave CNCs samples. FWHM, full width at half maximum.

	Crystalline Plane (Miller Index of I_β_)	FWHM (°)	Peak Position 2*θ* (°)	*d*-Spacing (nm)	Crystallite Size *τ* (nm)
CNCs Conventional	$1\;\bar{1}\;0$	1.7	15.0	0.59	4.8
	1 1 0	1.4	16.7	0.52	6.0
	1 0 2		21.0		
	2 0 0	1.5	22.9	0.39	5.6
	0 0 4		34.5		
CNCs Microwave	$1\;\bar{1}\;0$	1.7	14.7	0.60	5.0
	1 1 0	1.4	16.4	0.53	6.1
	1 0 2		20.6		
	2 0 0	1.5	22.6	0.39	5.7
	0 0 4		34.2		
